# Symmetrical palatal fibromatosis: An additional case report with immunohistochemical characterization

**DOI:** 10.4317/jced.57732

**Published:** 2021-04-01

**Authors:** Patrícia Caldeira, Karine da Silva, Ana-Carolina Vasconcelos, Giovanna Souto, Ricardo Mesquita

**Affiliations:** 1 DDS, MsC, PhD. Department of Oral Pathology and Surgery, School of Dentistry, Universidade Federal de Minas Gerais, Belo Horizonte, MG, Brazil; 2 DDS, MsC, PhD. Department of Semiology and Clinics, School of Dentistry, Universidade Federal de Pelotas, Pelotas, RS, Brazil; 3 DDS, MsC, PhD. Oral Pathology Section, School of Dentistry, Pontifical Catholic University of Minas Gerais - PUC Minas, Belo Horizonte, MG, Brazil

## Abstract

**Background:**

The term “symmetrical palatal fibromatosis” was recently suggested to designate bilateral palatal lesions presenting as typically broad, “mirror” images on the posterior lateral region of the hard palate.

**Purpose:**

We report an additional case of this as-yet poorly understood oral lesion in a 67-year-old male patient, with emphasis on differential diagnoses and immunohistochemical characterization.

**Case Report:**

The histopathological examination demonstrated a hypocellular, fibrous connective tissue with prominent thick collagen bundles and few blood vessels. Scattered large, stellate, and sometimes binucleated fibroblasts were found. Immunohistochemistry was positive for vimentin and negative for smooth muscle actin, S-100, desmin, HHF-35, AE1-AE3, Factor XIIIa, CD68, and FOSL1. This is the second study to show the immunohistochemical profile, with emphasis in FOSL1, of an additional case of symmetrical palatal fibromatosis.

**Conclusions:**

We encourage further reports about this entity, especially in relation to immunohistochemical and molecular features, so far poorly described, but very important for better recognition of this entity.

** Key words:**Palate, symmetrical palatal fibromatosis, desmoplastic fibroblastoma, immunohistochemistry.

## Introduction

Bilateral palate masses have historically been reported under different designation, such as bilateral fibroma of the palate, bilateral fibrous hyperplasia of the palate, symmetrical gingival fibromatosis, among others (1,2). Recently, Vargo *et al.* (1) suggested the term “symmetrical palatal fibromatosis” (SPF) to designate bilateral palatal lesions presenting as typically broad, “mirror” images on the posterior lateral surface of the hard palate. SPF may extend to the gingivae and/or crestal tuberosity, but its origin is from posterior lateral surfaces of the hard palate (1). Despite being an innocuous lesion, SPF can lead to dysfunctional speech, gagging, malocclusion, chewing, swallowing difficulties, and prosthesis displacement (1).

Microscopically, SPF mimic other fibrous-connective lesions, and the correlation between clinical, radiographical and histopathological features is necessary to their definitive diagnosis. The presence of fibrous, poorly vascularized, and hypocellular connective tissue with little or absent inflammatory infiltrate may also be found in fibromas, giant cell fibromas, gingival fibromatosis, and desmoplastic fibroblastoma (also called collagenous fibroma) (1-3).

Vasconcelos *et al.* (3) presented a case of a bilateral swelling in the hard palate, and named them as oral bilateral collagenous fibroma, an entity rarely reported in the mouth, even more bilaterally. As Vargo *et al.* (1), we believe the lesion reported by Vasconcelos *et al.* (3) represents a SPF. Therefore, it may be considered that Vasconcelos *et al.* (3) presented for the first time the immunohistochemical characterization of the SPF. However, the expression of the FOSL1 protein, which is diagnostic key to desmoplastic fibroblastoma, and could differentiate desmoplastic fibroblastoma from SPF was not performed. FOSL1 is overexpressed in desmoplastic fibroblastoma revealing a functional outcome of the 11q12 rearrangement found in this lesion (4,5).

Here, we report an additional case of SPF, offering a clinical, histopathological, and broad immunohistochemical characterization of this lesion.

## Case Report

**Figure 1:**
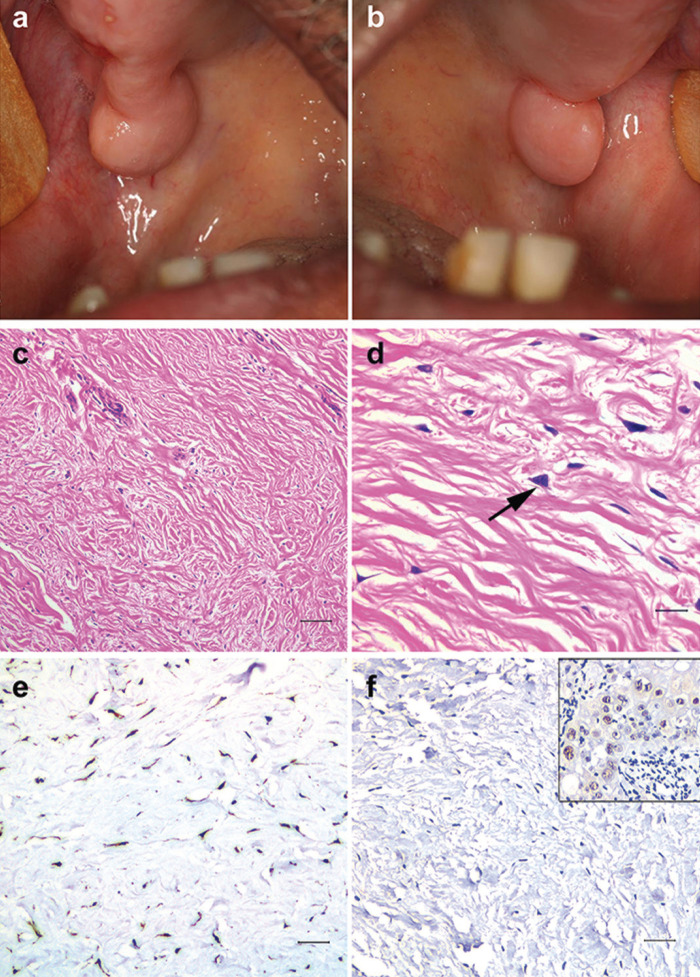
Clinical presentation. (a, b) Bilateral tumoral masses on posterior region of hard palate/tuberosity with well-defined margins and covered by healthy mucosa. Histopathology. (c) Hypocellular, fibrous connective tissue with prominent thick collagen bundles (scale bar: 80 µm). (d) Scattered large, stellate, binucleated fibroblasts (arrow) (scale bar: 20 µm). Immunohistochemistry. (e) Diffuse, strong positivity for vimentin and (f) negativity for FOSL1. Positive control to FOSL1 (in box) demonstrated positivity in the cell nucleus on squamous cell carcinoma (scale bar: 40 µm).

Written informed consent for patient information and images to be published was provided by the patient. The lesion was found in a 67-year-old male patient who sought the dental clinic for prosthetic rehabilitation. The intra-oral examination revealed bilateral tumoral masses with well-defined margins, covered by otherwise healthy mucosa and firm upon palpation (Fig. 1a,b). The lesions had a sessile insertion and were located bilaterally on the posterior surface of the hard palate/tuberosity. The evolution time was 1.5 years and the lesion was asymptomatic. The patient was edentulous and had never used dentures. Socioeconomic and familial histories were noncontributory. The panoramic radiograph revealed no bone involvement. Excisional biopsy was performed under local anesthesia and no recurrence was observed during three months of follow-up.

The histopathological examination revealed a hypocellular, fibrous connective tissue with prominent thick collagen bundles and few blood vessels (Fig. 1c). Scattered large, stellate, and sometimes binucleated fibroblasts were found (Fig. 1d). Chronic inflammatory cells were seldom. No myxoid change was found and the epithelial lining was regular. The immunohistochemical analysis revealed strong and diffuse positivity for vimentin (Fig. 1e) and negativity for smooth muscle actin, S-100, desmin, HHF-35, AE1-AE3, and FOSL1 (Fig. 1f). Scarce cells were positive for Factor XIIIa and CD68, but were infiltrating dendritic cells and macrophages, respectively, rather than lesion cells. Positive controls to all antibody were used (Fig. 1f-box).

## Discussion

In this report we present an additional case of SPF with characterization of its immunohistochemical profile with emphasis in the FOSL1 immunoexpression.

Vargo *et al.* (1) provided an updated literature review on SPF. The authors suggest that a previous report from our research group, entitled “ Oral bilateral collagenous fibroma: A previously unreported case and literature review “ at time of publication, would be better designated SPF (1,3). Considering the detailed clinical and histopathological characterization presented by Vargo *et al*. (1) for SPF, we agree with the suggestion. Accordingly, the paper by Vasconcelos *et al.* (3) would be the first study to provide the immunohistochemical features of this entity, revealing positivity to vimentin, α-smooth muscle actin and factor XIIIa, and negativity for S-100, CD68, CD34, HHF35, desmin and AE1/AE3. As mentioned before, the Factor XIIIa and CD68 immunopositivity found in the current case was interpreted as dendritic cells and macrophages. Additionally, the negativity of lesion cells to α-smooth muscle actin antibody is compatible with the findings of HHF-35 and desmin negativity, excluding muscle differentiation of lesion cells. Investigation of α-smooth muscle actin expression in further SPF cases would be helpful to differentiate it from desmoplastic fibroblastomas, since most cases of this entity show positivity for this immunomarker (3).

Importantly, Vargo *et al.* (1) pointed out the histological similarity between SPF and oral desmoplastic fibroblastoma (1,6). Intense, diffuse nuclear immunopositivity to FOSL1 was recently reported in desmoplastic fibroblastoma (non-oral lesions) but not for fibroma of tendon sheath or other spindle cell tumors (5). Therefore, FOSL1 was suggested as an aid for the diagnosis of desmoplastic fibroblastoma (5). The present case was tested for FOSL1 immunoreactivity, which proved negative. This is an additional finding supportive of the designation of SPF rather than desmoplastic fibroblastoma for the current case.

Clinically, the case reported here shows a broad-based insertion, following the pattern of a fibromatosis rather than a fibroma, as postulated by Vargo *et al.* (1). In addition, the absence of prosthetic trauma in the region, the lesion surface without erythema, and the few chronic inflammatory cells present in the specimen, altogether are supportive features of a non-reactive lesion (1).

Differential diagnosis with symmetrical gingival fibromatosis needs to be done. Symmetrical gingival fibromatosis represents a localized gingival fibromatosis, that in contrast to generalized gingival fibromatosis has no well-established hereditary nature, and no recurrence tendency (2). However, as opposed to SPF, symmetrical gingival fibromatosis normally appears as pedunculate masses, in young individuals (2). Histologically, symmetrical gingival fibromatosis has a myxomatous appearance, while SPF presents a densely collagenized swelling (1,2).

We look forward to further reports on SPF, especially with regards to its immunohistochemical and molecular features, for a better characterization of this as-yet poorly understood oral lesion.
